# Sulforaphane Modulates Cell Migration and Expression of β-Catenin and Epithelial Mesenchymal Transition Markers in Breast Cancer Cells

**Published:** 2020-01

**Authors:** Mehdi BAGHERI, Mozhgan FAZLI, Sara SAEEDNIA, Majid GHOLAMI KHARANAGH, Naghmeh AHMADIANKIA

**Affiliations:** 1 Clinical Research Development Unit, Imam Hossein Hospital, Shahroud University of Medical Sciences, Shahroud, Iran; 2 School of Medicine, Shahroud University of Medical Sciences, Shahroud, Iran; 3 Student Research Committee, School of Medicine, Shahroud University of Medical Sciences, Shahroud, Iran; 4 Cancer Prevention Research Center, Shahroud University of Medical Sciences, Shahroud, Iran

**Keywords:** Sulforaphane, Metastasis, Breast cancer, EMT, β-catenin

## Abstract

**Background:**

We aimed to assess the effect of sulforaphane (SFN) on breast cancer cell migration and also its effect on the expression of epithelial mesenchymal transition (EMT) markers and β-catenin.

**Methods:**

This study was performed in Shahroud University of Medical Sciences, Shahroud, Iran from 2017–2018. In this experimental study, MDA-MB-231 cells were treated with different concentrations of SFN (5, 10, 20, 30 and 40 μM) at different time points of 24, 48, and 72 h. The control group was untreated cells. The inhibitory effects of different concentrations of SFN on cell migration at different time points were evaluated using scratch assay. Moreover, apoptosis was assessed by using flow cytometric analysis. The expression of β-catenin and EMT markers of ZEB1, fibronectin, and claudin-1 were determined by real-time PCR. Western blotting analysis of β-catenin was applied to determine its changes after SFN treatment.

**Results:**

SFN markedly inhibited the migration of cells at concentrations of 10, 20, 30, and 40μM after 24, 48, and 72 h. At relatively, high concentrations (30, 40μM), SFN induced apoptosis. Moreover, SFN reduced the gene expression of ZEB1, fibronectin, and claudin-1 after 72 h. The expression of β-catenin revealed a time-dependent decrease at the concentration of 40 μM SFN.

**Conclusion:**

Downregulation of EMT markers and β-catenin showed accordance with the inhibition of migration. SFN could be a promising drug candidate to reduce metastasis in breast cancer.

## Introduction

Breast cancer is the most common cancer in women and the leading cause of cancer-related death among females worldwide. In fact, the cause of death in many patients with breast cancer is tumor spreading to other parts of body. Currently, there is not a cure for metastatic breast cancer and patients live approximately five years after initial diagnosis (1).

Metastasis is an enormously complex biological process involving different genes and biomolecules. More recently, epithelial-mesenchymal transition (EMT) has been shown to be one of the key regulators of cancer metastasis. EMT is a physiological process by which epithelial cells lose their adherent junctions and apical-basal cell polarity to form spindle-shaped cells that contribute to their ability to migrate as single cells. Loss of epithelial markers such as E-cadherin and acquisition of mesenchymal markers like fibronectin is a fundamental event in EMT. This switch in cell structure and behavior is mediated by key transcription repressors such as zinc finger proteins of ZEB family (2). Additionally, dysregulation of claudin-1 both increase and decrease in expression has been reported in several cancers (3). Moreover, upregulation of Wnt/β-catenin pathway has been demonstrated to play an important role in the transcription of EMT-promoting genes followed by cancer metastasis (4).

In recent years, much attention has been directed towards therapeutic strategies based on targeting β-catenin and EMT markers as the key players in cancer metastasis. There is a constant demand to develop less toxic, more efficacious, and affordable anticancer drugs with reduced side effects. In recent years, cancer prevention by natural products has received considerable attention(5). Among various natural products, sulforaphane (SFN), a chemopreventive is thiocyanate derived from broccoli, showed cancer inhibitory properties. SFN has been shown to inhibit cell cycle progression, induce apoptotic cell death, and inhibit angiogenesis in a variety of cancer cell types (6, 7).

Considering the promising anticancer properties of SFN, the aim of this study was to evaluate the effects of various concentrations of SFN on cell migration in MDA-MB-231 human metastatic breast cancer cells at different time points of 24, 48, and 72 h. Moreover, the expression of certain key elements of EMT, including ZEB1, fibronectin, and claudin-1 in breast cancer cells were examined in vitro after treatment with SFN. Furthermore, as upregulation of the Wnt/β-catenin signaling pathway has also been shown to lead to tumor metastasis, our present study was designed to determine the expression level ofβ-catenin in MDA-MB-231 breast cancer cells in response to SFN.

## Materials and Methods

### Cell culture

In this in vitro experimental study, human breast cancer cell line (MDA-MB-231), was obtained from the Pasteur Institute, National Cell Bank of Iran. The study was performed in Shahroud University of Medical Sciences, Shahroud, Iran from 2017–2018. The SFN was purchased from Sigma Company. Cells were cultured in Dulbecco modified Eagle’s medium (DMEM), supplemented with 10% fetal calf serum (FCS), and antibiotics (Penicillin 100 IU/ml, Streptomycin 100 μg/ml). Cells were incubated at 37 °C in a humidified atmosphere composed of 95% air and 5% CO2.

### Apoptosis assay

MDA-MB-231 cells were plated at a density of 2×10^5^ cells/well in six-well plates. Cells were treated with different concentrations of SFN (5, 10, 20, 30 and 40 μM). Untreated cells were considered as control group. After time points of 24, 48, and 72 h, the cells were trypsinized and washed with PBS. Annexin-V-FITC/PI labeling was performed according to the manufacturers’ instructions. Quantification of Annexin-V/propidium iodide incorporation was performed using a FACScalibur flow cytometer (BD Biosciences, San Jose, CA, USA). Acquired data were analyzed using the Win-MDI software.

### The cell scratch assay

The effect of SFN treatment on cell migration was determined using scratch assay as described previously (8). Briefly, a fine scratch was made on the surface of monolayer culture when the cells were approximately 80% confluent. A cell-free area of approximate 1 mm in extent was generated. Micrograph images from scratch zone were taken at time=0. Then cells were treated with 5, 10, 20, 30, and 40μM of SFN. Untreated cells were considered as control group. The choice of these doses was based on the previous study (9). The other micrographs were taken from the same region, after 24, 48 and, 72 h. The gap width of scratch was compared with the gap size at 0 h and analyzed by Image J software.

### RNA extraction and Real-time PCR

By using TriPure isolation reagent (Roche, Germany) based on the manufacturer’s instructions, total RNA was extracted from cells. The extracted RNAs were treated with DNase I enzyme to eliminate probable contamination with genomic DNA. Reverse transcription and real-time PCR were performed following the manufacturer’s instructions using SYBR Green Master Mix (Parstoos, Iran). Primers used in this study are shown in Table 1. The conditions for all reactions were as follows: denaturation at 95 °C for 10 min and 40 cycles of 95 °C for 15 sec, 60 °C for 30 sec, and 72 °C for 30 sec (10, 11).

**Table 1:** Real-time PCR primer sequences

***Primers***	***Sequence***	***Product size***
ZEB1	F: TGCACTGAGTGTGGAAAAGC R: TGGTGATGCTGAAAGAGACG	237 bp
Fibronectin	F: TCCTTGCTGGTATCATGGCAG R: AGACCCAGGCTTCTCATACTTGA	74 bp
Claudin-1	F: ACTCCTTGCTGAATCTGAACAGT R: GGACACAAAGATTGCGATCAG	97 bp
GAPDH	F: AAGGTGAAGGTCGGAGTCAAC R: GGGGTCATTGATGGCAACAATA	102 bp

### Western blotting analysis

The pellets of control and treated cells after the proposed time points of 24, 48, and 72 h were suspended in lysis buffer (BioBasic, PH= 8), supplemented with protease inhibitor cocktail (Sigma-Aldrich). Forty micrograms of proteins were denatured using Laemmli’s sample Buffer and boiled at 95 °C for 5 min. Proteins were separated by SDS-polyacrylamide gel electrophoresis and transferred onto PVDF membranes (Amersham). The membranes were placed in a blocking buffer for 60 min at RT and subsequently incubated with anti-β-catenin primary antibody (1:1000; Cell Signaling) overnight, at 4 °C. The following day, the membranes were washed and incubated with a peroxidase conjugate secondary antibody (1:5000; Cell Signaling) for 60 min, at RT. Then membranes were washed and incubated with the Bio-Rad Clarity™ western ECL substrate and finally exposed to X-ray film from FUJIFILM Corporation. Band intensity was calculated using Image J software.

### Statistical analysis

The statistical analyses were performed using GraphPad Prism version 6.0 software. One way ANOVA with Dunnett's multiple comparison test was used to assess differences between various groups. Data are presented as mean ± standard deviation (SD).

## Results

### SFN inhibited the migration of breast cancer cells

**Fig. 1:**
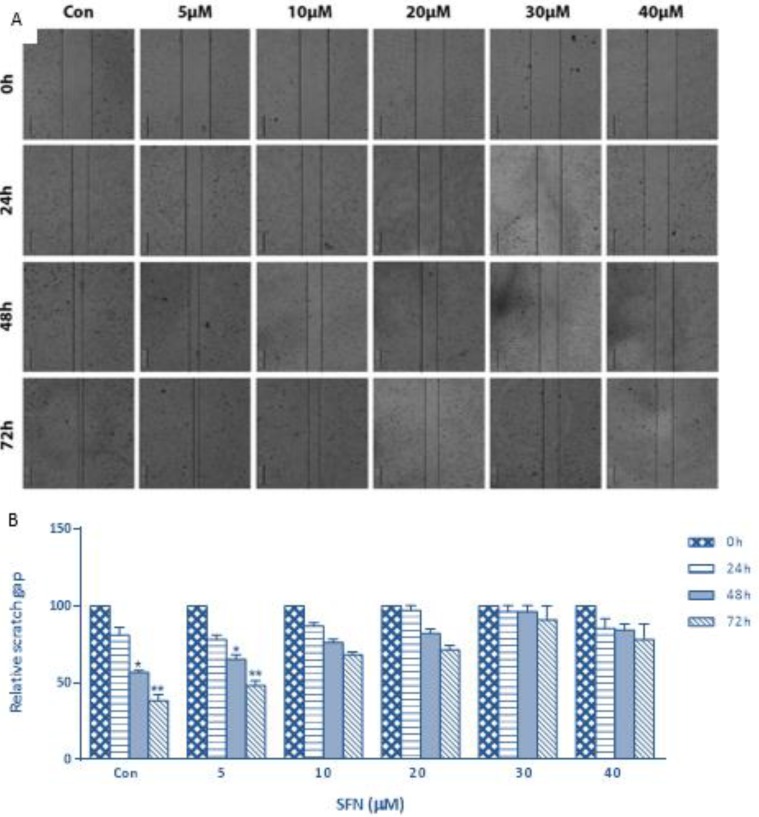
The migration capability of SFN treated MDA-MB-231 cells was evaluated by scratch assay. (A) Scratch assay images show the extent of healing and black lines indicate the scratch borders. Scale bar: 200 *μ*m. (B) Graph represents statistical analysis of scratch assay. Data show a role of the SFN as a suppressor of breast cancer cell migration. Significance was set at ^*^P< 0.05, ^**^P< 0.01

To investigate the effect of SFN on cell metastatic potential, the migration capacity was detected using scratch assay. SFN suppressed migration of cells to the denuded zone at concentrations of 10, 20, 30, and 40 μM (Fig. 1 A, B). These findings provide evidence for the role of the SFN as a suppressor of breast cancer cell migration.

### SFN induced cell apoptosis in MDA-MB-231 Cells

**Fig. 2:**
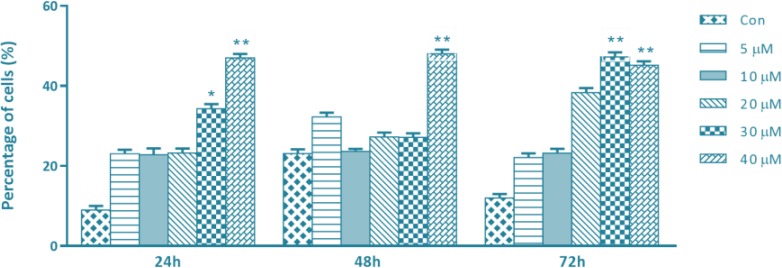
SFN enhanced apoptosis in breast cancer cells. Human breast cancer MDA-MB 231 cells were treated with increasing concentrations of SFN (0–40 μM) for 24, 48 and 72 h. The flow cytometry assay was used to assess cell viability and the results were normalized to the untreated control cells. Quantitative data show a significant increase of apoptosis at 30 and 40 μM of SFN in compare with control cells. ^*^ and ^**^ donate *P*<0.05 and *P*<0.01 respectively, as compared with the control

The ability of SFN to induce apoptosis was assessed by flow cytometry analysis. MDA-MB-231 cells were treated with different concentrations of SFN (0, 5, 10, 20, 30 and 40 μM) for 24, 48 and 72 h. As shown in Fig. 2, compared with untreated cells, SFN increased the percentage of the early/late apoptotic and necrotic cells significantly, at the maximum doses of 30 and 40 μM.

### SFN decreased expression of genes involved in EMT

**Fig. 3:**
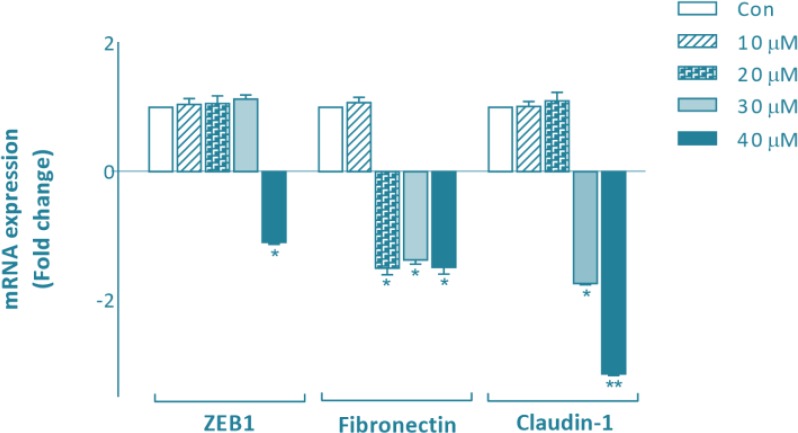
Effects of SFN on transcriptional levels of specific genes involved in EMT. Total RNA was extracted from MDA-MB-231 cells treated for 24, 48, and 72 h with 10, 20, 30 and 40μM of SFN. Various concentrations of SFN have different effects on the expression of EMT markers of ZEB1, fibronectin, and claudin-1 after 72 h. Fold changes in gene expression were analyzed relative to gene expression in untreated controls. Significance was set at ^*^P<0.05 and ^**^P<0.01

We performed quantitative RT-PCR to examine the effect of SFN on expression levels of specific genes involved in EMT such as ZEB1, fibronectin and claudin-1. In the present study, MDA-MB-231 cells were treated with different concentrations of SFN (0, 5, 10, 20, 30 and 40μM). Total RNAs were collected at 24, 48 and 72 h. We found that SFN significantly downregulated the expression of ZEB1 at 40μM after 72 h compared with the control group. SFN also decreased the expression of fibronection at 20, 30, and 40 μM after 72 h. Decreased expression of claudin-1 was observed at 30 and 40μM after 72 h (Fig. 3).

### SFN inhibited β-catenin expression in MDA-MB-231 cells

**Fig 4:**
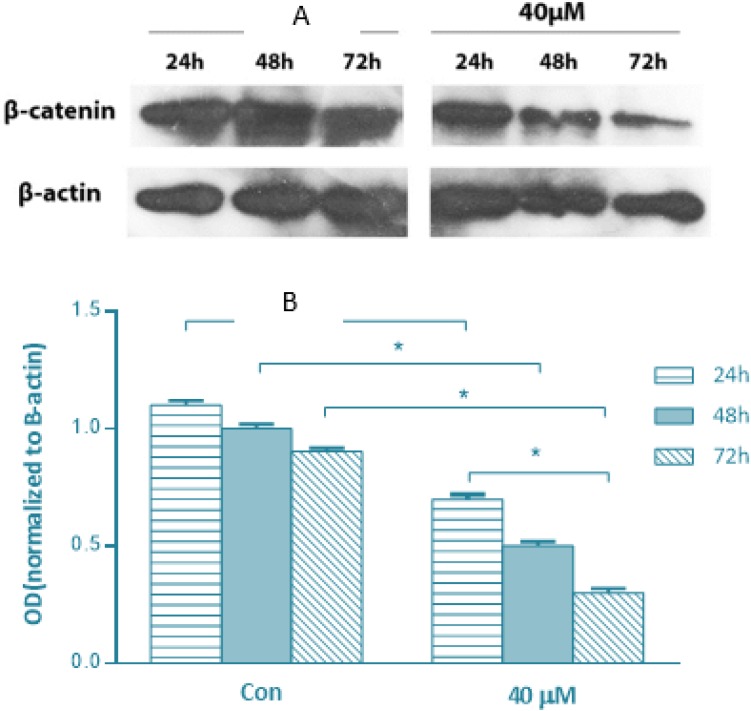
Immunoblot analysis of β-catenin with protein extracts from MDA-MB-231 cells treated with 40μM SFN for 24, 48 and 72 h compared with untreated control. (A) A representative original blot, (B) Quantitative data. SFN decreases β-catenin expression in breast cancer cells. Significance was set at ^*^P<0.05

To further investigate the possible mechanisms by which SFN exerts its anti-migratory effect, we evaluated whether the expression of β-catenin is associated with SFN treatment. Immunoblot analysis was employed to examine the expression of β-catenin. As shown in Fig. 4, there was a time-dependent decrease at the concentration of 40 μ MSFN. There were no changes in the expression of β-catenin protein in MDA-MB-231 cells with lower doses of SFN (data not shown).

## Discussion

The present study illustrates that SFN exposure induces apoptosis at 30–40μMand also inhibits the migratory capabilities of MDA-MB-231 cells at 10–40μM. These results raise the possibility that SFN may be a promising candidate to prevent breast cancer progression and metastasis. It was revealed that the anti-cancer activity of SFN is modulated by a variety of cell-signaling pathways including NF-κB signaling (12), Keap1– Nrf2 signaling (13), and ROS-dependent pathway (14). Further studies are needed to investigate the underlying molecular mechanisms of SFN effects to provide a rational basis for its clinical application in the future.

EMT is a crucial step for tumor cell migration and invasion in various types of human cancers. Overexpression of ZEB1, as a transcription factor, is critical for induction of EMT(15, 16). The results of our study revealed that SFN decreased cell migration at concentrations of 10–40μMSFN; however, reduced expression of ZEB1was observed just at 40μM of SFN. It can be assumed that ZEB1 is not involved in mediating the anti-metastatic effect of SFN at lower concentrations in MDA-MB-231 cells. Recently, expression of ZEB1 activates signaling pathways leading to enhanced cell proliferation, and tumor growth (17). Based on the present study, SFN at high concentration may at least partly exerts its proapoptotic and antimigratory effects by targeting the expression of ZEB1 in MDA-MB-231 cells.

Fibronectin is another established EMT marker, which appears to be involved in extracellular matrix (ECM) remodeling during EMT. It is upregulated during development and has been linked to the growth, survival and metastasis in variety of cancers including breast (18), prostate (19) and colon (20) cancers. In accordance with previous studies, we find that decreased expression of fibronectin coincides with decreased cell migration in response to SFN treatment (10–40μM). Considering the important role of fibronectin in cancer metastasis, it is possible that SFN exerts its anti migratory effects by decreasing the expression of fibronectin. A more detailed study is required to clarify the exact role of fibronectin in this effect.

Moreover, claudins, the integral membrane proteins which form the backbone of tight junctions, are aberrantly expressed in diverse types of human cancers (21). Claudin-1, the first claudin family member identified, is downregulated in hepatocellular carcinoma (22), breast cancer (23) and lung adenocarcinomas (24). Conversely, overexpression of claudin-1 increases cell invasion in oral squamous cell carcinoma (25), melanoma (26), colon (27), and colorectal cancers (28). Further support for such speculations comes from studies that showed increased expression of claudin-1 induced expression of the EMT-regulating transcription factors such as ZEB1 in hepatocellular carcinomas, and claudin-1 can be exploited as a biomarker for liver cancer metastasis (29). Collectively, claudin-1 can both promote or suppress tumorigenesis, depending on the cancer type.MDA-MB-231, the cell line used in this study, is a basal B/triple-negative breast cancer (TNBC) cell line (30, 31). The knockdown of claudin-1 in a basal-like subtype human breast cancer cell line resulted in reduced cancer cell migration (32). Furthermore, down-regulation of claudin-1 induced apoptosis in different breast cancer cells including MDA-MB-231(33). In accordance with these studies, results presented here indicated that decreased migration and increased apoptosis in MDA-MB-231 cells in response to SFN (30–40μM) coincided with reduced expression of claudin-1. Apoptotic and anti-metastatic effects of SFN at 30–40μMcan be at least partly mediated by decreased expression of claudin-1. Although cell migration decreased at concentrations less than 30 μM of SFN; however, it did not change the expression of claudin-1. Claudin-1 is not involved in antimigratory effect of SFN at low concentrations.

Additionally, several pieces of evidence suggest that Wnt/β-catenin signaling pathway plays critical role in tumor progression (34). In the absence of Wnt stimulation, cytosolic β-catenin is sequentially phosphorylated and degraded by glycogen synthase kinase 3β (GSK3-β) within a protein complex (35). Wnt stimulation is suggested to inhibit β-catenin phosphorylation, thus inducing the accumulation of cytosolic β-catenin. Next, the stabilized β-catenin is translocated into the nucleus, in which it activates the expression of a range of genes related to cancer progression and metastasis (36). Aberrant activation of Wnt/β-catenin pathway occurs in several tumor types, including colorectal and breast cancers (37, 38). ”Recently, **t**here is considerable interest in developing Wnt/β-catenin pathway inhibitors as anti-cancer therapeutics (39)”. In the current study, the effect of SFN on β-catenin, as the key mediator of the Wnt pathway in human breast cancer cells was examined. The results **of** our survey showed that SFN at 40μMsuppressed the expression of β-catenin in a time-dependent manner in breast cancer MDA-MB-231 cells. No change in the expression of β-catenin was observed at lower concentrations of SFN. Apoptotic and anti-metastatic effects of SFN at high concentration might be at least partly mediated through its effect on the expression of β-catenin. A casual mechanism of this association should be unraveled by more detailed study.

## Conclusion

SFN decreases migration of breast cancer cells and it can be considered as a promising candidate for treatment of metastatic breast cancer. Furthermore, anti-migratory effects of SFN at low concentrations may be mediated in part by decreased expression of fibronectin, not ZEB1, claudin-1 and β-catenin. Further attempts have to be made to understand more about the mechanisms involved in anti-metastatic effects of SFN on breast cancer cells.

## Ethical considerations

Ethical issues (Including plagiarism, Informed Consent, misconduct, data fabrication and/or falsification, double publication and/or submission, redundancy, etc.) have been completely observed by the authors.
